# Electronic health records to capture primary outcome measures: two case studies in HIV prevention research

**DOI:** 10.1186/s13063-023-07264-6

**Published:** 2023-03-31

**Authors:** David Dunn, Leanne McCabe, Ellen White, Valerie Delpech, Peter D. Kirwan, Jameel Khawam, Sara Croxford, Denise Ward, Elizabeth Brodnicki, Alison Rodger, Sheena McCormack

**Affiliations:** 1 MRC Clinical Trials Unit at UCL, London, UK; 2 Institute for Global Health, University College London, London, UK; 3 National Infection Service, UKHSA, London, UK

**Keywords:** Electronic health records, Routinely collected health-care data, Primary outcome, HIV

## Abstract

**Background:**

There is increasing interest in the use of electronic health records (EHRs) to improve the efficiency and cost-effectiveness of clinical trials, including the capture of outcome measures.

**Main text:**

We describe our experience of using EHRs to capture the primary outcome measure — HIV infection or the diagnosis of HIV infection — in two randomised HIV prevention trials conducted in the UK. PROUD was a clinic-based trial evaluating pre-exposure prophylaxis (PrEP), and SELPHI was an internet-based trial evaluating HIV self-testing kits. The EHR was the national database of HIV diagnoses in the UK, curated by the UK Health Security Agency (UKHSA). In PROUD, linkage to the UKHSA database was performed at the end of the trial and identified five primary outcomes in addition to the 30 outcomes diagnosed by the participating clinics. Linkage also produced an additional 345 person-years follow-up, an increase of 27% over clinic-based follow-up. In SELPHI, new HIV diagnoses were primarily identified via UKHSA linkage, complemented by participant self-report through internet surveys. Rates of survey completion were low, and only 14 of the 33 new diagnoses recorded in the UKHSA database were also self-reported. Thus UKHSA linkage was essential for capturing HIV diagnoses and the successful conduct of the trial.

**Conclusions:**

Our experience of using the UKHSA database of HIV diagnoses as a source of primary outcomes in two randomised trials in the field of HIV prevention was highly favourable and encourages the use of a similar approach in future trials in this disease area.

## Introduction

There is increasing interest in the use of electronichealth records (EHRs), also known as routinely collected health-care data, to improve the efficiency and cost-effectiveness of clinical trials [1–4]. EHRs can potentially be used to assess study feasibility, to facilitate recruitment, and lower the cost of data collection and follow-up visits. The US Food and Drug Administration (FDA) and the UK Medicines and Healthcare products Regulatory Agency (MHRA) have advocated the use of hybrid designs, in which pragmatic design elements to collect real-world data are built into traditional randomised controlled trials [5, 6]. However, the adoption of these approaches remains relatively low in practice [7, 8].

Here we describe our experience of using EHRs to capture the primary outcome measure — HIV infection or the diagnosis of HIV infection — in two contrasting HIV prevention trials in the UK. PROUD was a clinic-based trial evaluating pre-exposure prophylaxis (PrEP), and SELPHI was an internet-based trial evaluating HIV self-testing kits [9, 10]. The EHR in question was the national database of HIV diagnoses in the UK, curated by the UK Health Security Agency (UKHSA) [11]. In PROUD, linkage to the UKHSA database was used to complement data collected and reported by the participating clinics, whereas in SELPHI this was the principal data source for the primary outcome measure.

## Electronic health records of HIV diagnoses

**Fig. 1:**
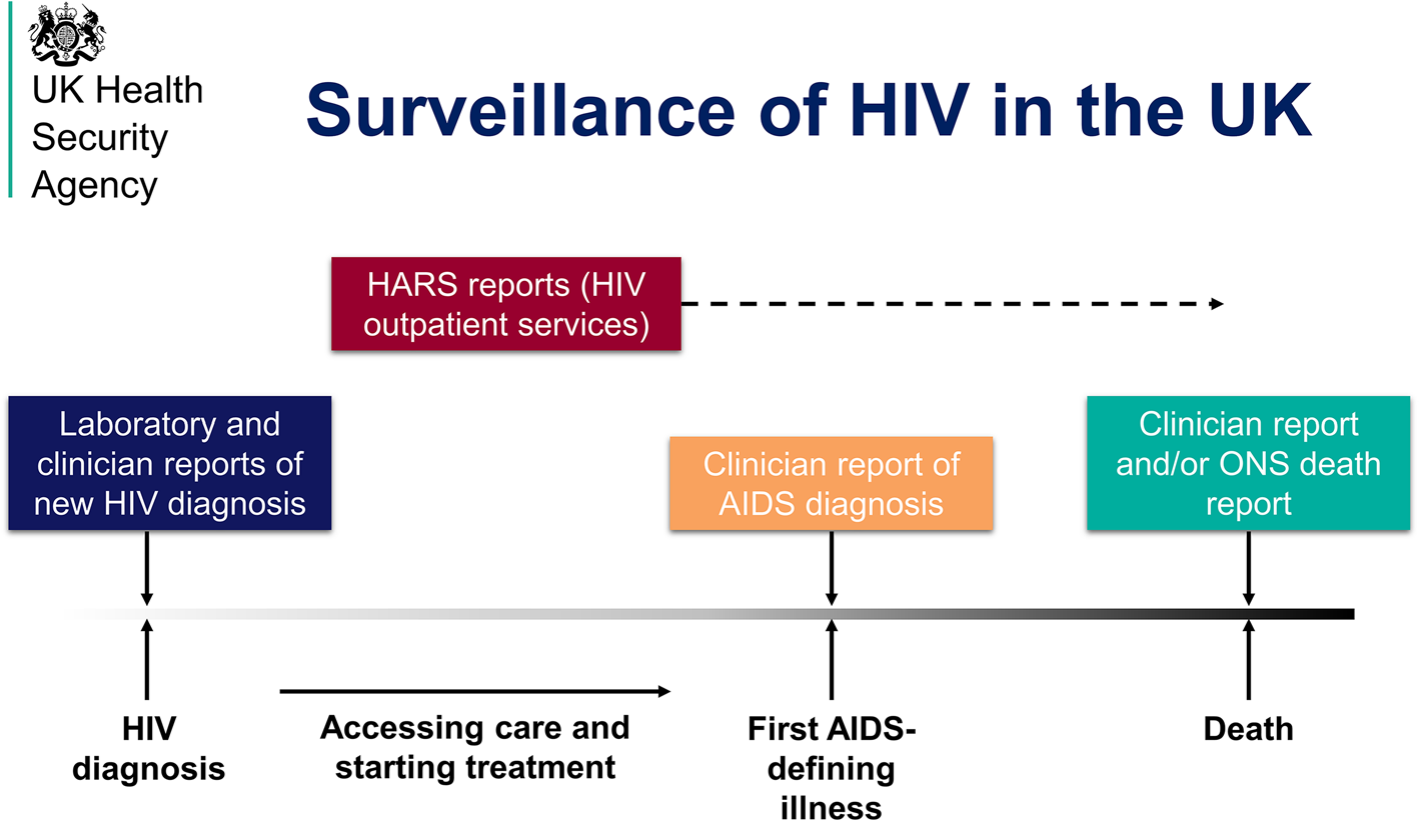
Schema of the HIV surveillance system in the UK

The UK Health Security Agency (UKHSA), known before October 2021 as Public Health England (PHE), collects pseudonymized case reports of confirmed new HIV diagnoses in England, Wales and Northern Ireland (EW&NI), which are combined annually with case reports collected by Public Health Scotland (PHS) to form a de-duplicated database of all new HIV diagnoses in the UK [11]. Most diagnoses are identified from submissions by outpatient HIV service providers in England, who report every 3 months to the HIV and AIDS Reporting System (HARS) at UKHSA. Reports of confirmatory diagnoses are also received from laboratories performing HIV testing data for General Practices and hospitals, HIV testing of pregnant women in antenatal care, and partner notification schemes, in addition to emergency care and community HIV testing settings. The data from these disparate sources are consolidated and de-duplicated, with a definitive dataset produced in the third quarter of each year that includes all HIV diagnoses that occurred within the previous calendar year. Figure 1 depicts a broad overview of the HIV surveillance system in the UK.

**Fig. 2:**
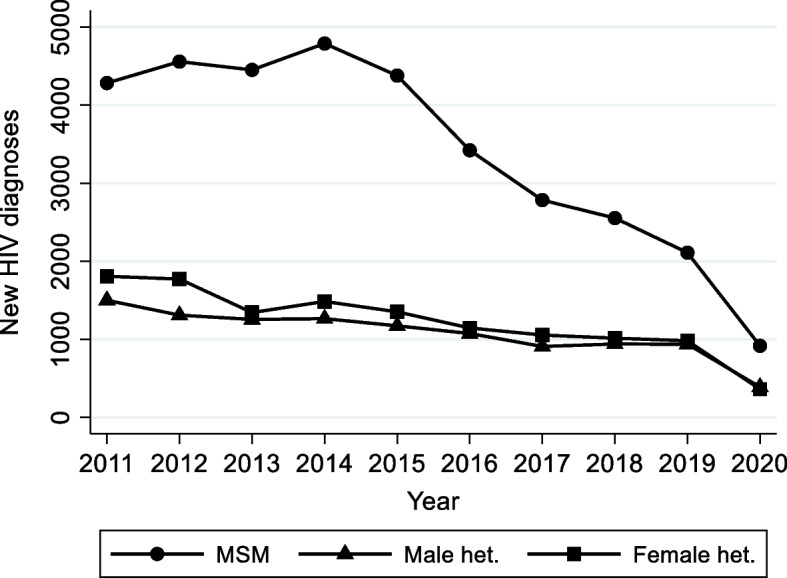
New HIV diagnoses in England by probable HIV exposure. Footnote: Data for graph obtained at https://www.gov.uk/government/statistics/hiv-annual-data-tables

Annual reports on the epidemiology of HIV infection in the UK are produced from this dataset, which provide important insights on the effect of preventative public health measures [12]. Focussing on England, the number of annual new diagnoses among men who have sex with men (MSM) has declined steadily since 2014, falling approximately fivefold to slightly over 900 in 2020 (Fig. 2). This is thought to reflect intensified HIV testing combined with immediately received anti-retroviral therapy and roll-out of PrEP [12]. The number of diagnoses has also declined, although more gradually, in those who likely acquired HIV infection through heterosexual contact (both males and females). The sharp drop in the number of cases in 2020 is attributable to the COVID-19 pandemic, which changed patterns of both sexual behaviour and HIV testing.

## PROUD trial

### Background

The PROUD trial was an open-label RCT to evaluate the *effectiveness *of daily oral pre-exposure prophylaxis (PrEP) to prevent HIV infection (ISRCTN94465371) [9]. Previous placebo-controlled trials had confirmed the high biological *efficac*y of PrEP, but prior to PROUD concern existed that users knowingly taking PrEP would increase risky sexual behaviour and negate the biological protection conferred by PrEP. Participants in PROUD were randomised to receive PrEP immediately (IMM group) or after a deferral period of 12 months (DEF group). A total of 545 MSM were enrolled from 13 sexual health clinics in England between November 2012 and April 2014. Eligible participants were male at birth, aged ≥ 18 years, had tested HIV negative in the previous 4 weeks or on the day of enrolment, and had reported anal intercourse without a condom in the previous 90 days.

**Fig. 3:**
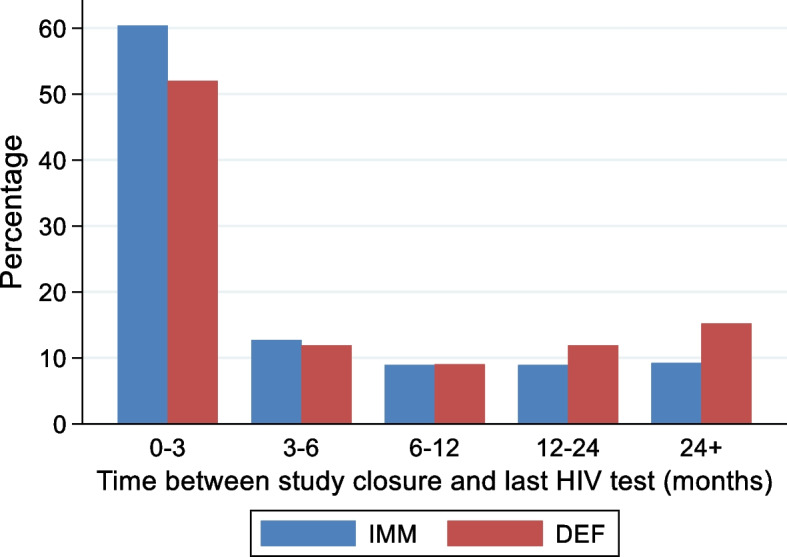
Time between study closure and last reported HIV test in PROUD. Footnote: Graphs exclude the 37 participants who acquired HIV infection or died

The primary outcome measure was defined as a confirmed HIV infection acquired within 12 months of randomisation (the “deferred phase”). Trial follow-up continued beyond 12 months until the 28 October 2016 (the “post-deferred phase”). This allowed a before-after comparison of access to PrEP in the DEF group and an examination of longer-term adherence to PrEP. Follow-up comprised clinic visits every three months, which included HIV and STI tests, PrEP dispensing (if appropriate), and safety monitoring. HIV diagnoses were reported to the coordinating centre in real-time by the participating clinics. At the end of the trial, this information was supplemented by matching to the UKHSA HIV diagnoses database (see Appendix for details). This had two main benefits: first, to capture diagnoses made in non-participating clinics and/or other settings; second, to increase person-years of observation since there was an appreciable loss to follow-up, particularly in the DEF group. The interval between the date reported earlier and the last reported HIV test exceeded 12 months for 18% of participants in the IMM group and 27% of participants in the DEF group (Fig. 3).

### Primary outcome results

Incident HIV infection was diagnosed in a total of 30 individuals by participating clinics. Reassuringly, all 30 cases were also identified in the UKHSA database, and matching identified an additional six previously unrecognised HIV diagnoses. Liaison with participating clinics confirmed that five of these were genuine PROUD participants but that one was a spurious match (matched on date of birth and initial, did not match on surname Soundex). Four of the additional diagnoses occurred during the post-deferred phase and one during the deferred phase.

HIV incidence rates were calculated with and without incorporating information from the UKHSA matching, which affects both denominators and numerators (Table 1). For the analysis *with* UKSHA matching, administrative censoring was used, namely the date of trial closure (28 October 2016). This implicitly assumes that all HIV diagnoses prior to this date would have been reported to UKHSA when matching was performed in September 2017. For the analysis *without* UKHSA matching, the censoring date was the latest HIV test performed in a participating clinic.

**Table 1:** HIV diagnoses in PROUD: impact of including information from UKHSA matching

Phase of trial	Allocated group	HIV diagnoses ascertained by clinics only			HIV diagnoses ascertained by clinics or via UKHSA matching		
		**Cases**	**PYFU**	**Incidence rate (95% CI)**	**Cases**	**PYFU**	**Incidence rate (95% CI)**
Deferred	DEF	20	224.2	8.9 (5.6–13.5)	21	264.8	7.9 (5.0–11.0)
	IMM	4	254.4	1.6 (0.5–3.8)	4	270.5	1.5 (0.5–3.6)
Post-deferred	DEF	1	357.3	0.3 (0.0–1.4)	3	511.0	0.6 (0.1–1.6)
	IMM	5	426.7	1.2 (0.4–2.6)	7	560.9	1.2 (0.5–2.5)

Confidence intervals derived by exact mid-*P* value method

*PYFU*, person-years of follow-up

Incidence rates expressed per 100 PFYU

Inclusion of information from UKHSA matching produced an additional 56.7 person-years follow-up for the deferred phase (11.8% increase) and an additional 287.9 person-years follow-up for the post-deferred phase (36.7% increase) (Table 1). HIV incidence estimates changed only marginally, although the extra data resulted in narrower confidence intervals. Two main substantive conclusions can be drawn. First, the dramatic reduction in HIV incidence in the DEF group after they were offered PrEP confirms the findings of the primary randomised analysis, which compared the IMM and DEF groups during the deferred phase [9]. Second, the low incidence in the IMM group was maintained for the duration of follow-up (median 3.0 years), refuting prior concerns that good adherence to PrEP would be transient.

## SELPHI trial

### Background

SELPHI was an internet-based, randomised controlled trial which assessed whether providing free HIV self-testing (HIVST) kits led to earlier diagnosis of HIV infection (ISRCTN20312003) [13]. The trial had a two-stage randomisation. In the first randomisation, participants were randomised (in a 3:2 ratio) to receive (BT group) or not receive (nBT group) a free, single HIVST kit; the rationale was to identify prevalent, unrecognised HIV infections. In the second randomisation, HIV-negative participants at high risk of incident HIV infection were randomised (in a 1:1 ratio) to the offer of regular (every 3 months) free HIVST kits; the rationale was to reduce the average interval between the acquisition and diagnosis of HIV infection. The current paper focusses on the first randomisation [10].

A total of 10,791 participants were enrolled between February 2017 and March 2018 via adverts placed on various internet sexual and social networking sites. Eligible participants (based on self-report) were men (including trans-men), aged ≥ 16 years, resident in England or Wales, ever had anal intercourse with a man, not having a positive HIV diagnosis, and being willing to provide name, email address, date of birth, and consent to link to national HIV databases.

The primary outcome measure was a confirmed new HIV diagnosis within 3 months after randomisation. Online surveys collected data at baseline, 2 weeks (BT group only), 3 months, and at study closure. At each follow-up survey, participants allocated to BT were asked about their experience of using the HIVST kit, including the result of the test. At the 3-month and final surveys, participants in both groups were asked if they had had any positive HIV tests (other than the SELPHI HIVST kit). However, response rates to the surveys were low: 67% and 39% in the BT and nBT groups, respectively, at the 3-month survey, and 44% and 26%, respectively, at the final survey. UKHSA linkage was therefore the main source of information on the primary outcome measure; linkage was performed on a regular basis, approximately every 3 months, throughout the trial.

### Primary outcome results

Table 2 shows the concordance between self-reported HIV diagnoses and those recorded in the UKHSA database. Thirty-three cases were recorded in the UKHSA database, of which only 14 had also been self-reported. Three participants reported a positive HIV test but did not link to the UKHSA database. The study clinicians made up to three attempts to contact these participants to clarify the reason for this. One was confirmed as having linked to care, but the two other participants did not respond to email requests for an offer to be seen in clinic. It is not possible to ascertain if this represents a failure of linkage to care or incorrect reporting of a positive HIV test. This gives a total of 34 participants who experienced the primary outcome.

**Table 2:** HIV diagnoses within 3 months after randomisation in SELPHI: concordance between self-reported diagnoses and UKHSA database

Self-reported diagnosis	Diagnosis in the UKHSA database	
	No	Yes
No — did not complete 3-month survey	-	17^a^
No — completed 3-month survey but did not report a positive test	-	2
Yes	3^b^	14

^a^One of these cases was reported at the final survey

^b^1 case was confirmed as having linked to clinical care, and 2 cases were unconfirmed. The two unconfirmed cases did not contribute to the main primary outcome analysis

UKHSA linkage played another important role in the analysis. Although all participants declared that they had never tested HIV positive on the enrolment survey, 89 participants were subsequently identified as matching to the UKHSA database with a date of diagnosis *before *the date of enrolment. Many of these diagnoses occurred several years earlier, with a median (IQR) interval of 60 (18,124) months. Although some of these matches may have been spurious we adopted the conservative approach of excluding all 89 participants from analyses since this resulted in relatively little loss of information. An additional 556 participants were excluded for other reasons (mostly duplicate enrolments), leaving 10,111 (6049 BT, 4062 nBT) in the final analysis [10].

Of the 34 confirmed HIV diagnoses within 3 months, 19 (0.3%) were in the BT group and 15 (0.4%) in the nBT group, a risk difference of − 0.1% (95% CI − 0.3%, 0.2%). Thus SELPHI provided no evidence that offering a single free HIVST kit increased rates of HIV diagnosis, despite much higher HIV testing rates in the BT group [10]. However, the trial was statistically under-powered as the observed diagnosis rates were much lower than assumed in the sample size calculation (between 1.25% and 2.0%) [13].

## Discussion

Our experience of using EHRs to identify primary outcome measures in two randomised HIV prevention trials was highly favourable. Although the same primary outcome measure was used in the two trials, their aims were subtly different: PROUD aimed to prevent the acquisition of HIV infection per se, whereas SELPHI aimed to increase the rate of HIV diagnoses. In the PROUD study, information from UKHSA linkage complemented the data collected in participating clinics. A similar hybrid approach has been used in trials in other areas of research [14, 15]. It is noted that the trial could not have depended exclusively on UKHSA linkage for capturing the primary outcome measure — it may take up to one year for a new HIV diagnosis to be reported and consolidated in the UKHSA database, whereas the Independent Data and Monitoring Commitee reviewed emerging data on a monthly basis, given the ethically sensitive nature of the trial [9]. In contrast, SELPHI was less time-sensitive, and UKHSA linkage was essential for capturing HIV diagnoses and thus the successful conduct of the trial.

As commonly occurs in prevention trials, rates of loss to follow-up in PROUD were relatively high since participants were generally healthy and had little incentive to attend clinic other than to get their repeat PrEP prescription. Loss to follow-up may well have been related to the risk of acquiring HIV infection (i.e. informative censoring) [16]; for example, as participants who were no longer having risky sex are more likely to discontinue PrEP. This underscores the importance of UKHSA linkage for obtaining unbiased, robust estimates of HIV incidence. Informative loss to follow-up is a potentially important problem in other open-label extension studies of PrEP trials, but the issue has generally not been addressed [17].

In SELPHI, survey response rates were low — 67% in the BT group and 39% in the nBT group at the key survey at 3 months. However, these figures are not unusually low for internet-based studies of HIV self-testing [18]. Reliance on self-reported data alone would have missed over half of the newly diagnosed cases, and comparisons between the randomised groups would be seriously biased due to the different response rates. UKHSA linkage also revealed a large number of participants who were already HIV positive at enrolment and thus not eligible for the trial. The inclusion of these cases, which dwarfed the number of genuine primary outcome events, would have seriously distorted the trial findings. The reason for these participants joining the trial is not known: they may have been curious to try out a self-test, they may have procured it for a friend, or they may have wanted to verify that they were still antibody positive (possibly confusing this with an undetectable viral load). Regardless of the reason, this is a cautionary lesson for studies in which inclusion criteria are assessed on self-reported data.

The valid use of EHRs depends critically on linkage between the patient identifiers held in the trial database and the EHR database. Given the sensitivity around HIV infection, pseudonymized identifiers were used in the PROUD and SELPHI trials. The identifiers in PROUD were provided by the clinic whereas in SELPHI they were provided online by the participants. The latter is much more prone to inaccuracy (either deliberate falsification or typographical errors) and is beyond the control of the study investigators. Some other practical issues merit comment. First, as data protection legislation in most countries requires the need for participant consent for linkage, it is important to include this information in the patient information sheet [19]. Second, consideration needs to be given as to how and where linkage takes place. The sensitive nature of the data precluded the export of the UKHSA HIV diagnoses database to the trial coordinating team. Instead, the trial statisticians provided UKHSA scientists with files containing participant identifiers, who then performed the linkage and returned pre-agreed information (most importantly, date of HIV diagnosis) on participants deemed to have matched. Another model we considered was to grant the trial statisticians supervised access to the UKHSA database, but all databases have intricacies that are usually best understood by the scientists working directly on them. The MHRA have recently provided guidance on system security on research access to EHR systems [6]. Finally, there are potential logistical considerations with the use of EHR for clinical trials, including specific ethics requirements for access to EHR datasets and additional costs for access and/or data linkage when performed by the data custodian.

As well as reliable linkage, the completeness and accuracy of the relevant data in the EHR is critically important [20]. Data incompleteness has been cited as a key reason for the relatively low adoption of EHRs in clinical trial research [21, 22]. A detailed process was recently described for assessing the integrity of the two most utilised UK NHS Digital data assets: the Admitted Patient Care dataset of Hospital Episode Statistics (HES APC) and the Civil Registration of Deaths (CRD) [4]. Part of this process involved the examination of the methods by which the datasets are produced, starting with the origins of the data. The authors of this report strongly encourage collators of EHR databases to systematically document their processes and for researchers to justify the validity of EHR-derived data in the trial protocol. The UKHSA HIV diagnosis database has not been subject to a formal external review. However, rigorous processes have been developed and refined over the 40 years since the onset of the HIV epidemic, including the triangulation of multiple data sources to minimise the risk of missing new diagnoses [11].

## Data Availability

See original main trial papers for data availability.

## Appendix

### Details of the matching processes

For each study, matching was based on a number of personal identifiers (described below for each study). These data items were sent securely from the coordinating centre (MRC CTU at UCL) to UKHSA, where the linkage was performed. To maintain blinding, the randomised allocation was not sent. Matching was performed using a deterministic, hierarchical algorithm written in STATA. The underlying logic of the algorithm was as follows:

- 1. Make a list of the matching criteria
- 2. Compare every record in PROUD/SELPHI to every record in the UKHSA database and pull out those records that have a match based on the criteria in step 1
- 3. Check the matches and repeat steps 1 and 2 a number of times with ever looser criteria until confident that all possible matches are being found
- 4. Manually review all the matches and group these into lists based upon the matching criteria (definite/review details/review date/not a match)

### PROUD

Matching by UKHSA was performed twice (September 2016, September 2017), based on Soundex (encoded surname), full initials, date of birth, clinic number, PROUD site, and last date of HIV screen in PROUD. The datasets included the 33 participants whose HIV infection (prevalent at baseline or incident) had been diagnosed by a clinic (although these were not flagged in the dataset). Reassuringly, all 33 were successfully matched by UKHSA, attesting to the sensitivity of the matching algorithm.

### SELPHI

Matching by UKHSA was performed approximately every 3 months throughout the trial based on full Soundex, first-name Soundex (F_ Soundex), initial, gender, date of birth (DOB), partial postcode (LSOA), ethnicity, and country of birth (COB). The matching hierarchy was as follows:

- 1. Soundex, Initial, Gender, DOB, LSOA, Ethnicity, COB
- 2. F_ Soundex, Initial, Gender, DOB, LSOA, Ethnicity, COB
- 3. Soundex, Initial, Gender, DOB, Ethnicity, COB
- 4. F_ Soundex, Initial, Gender, DOB, Ethnicity, COB
- 5. Soundex, Initial, Gender, DOB, Ethnicity
- 6. F_ Soundex, Initial, Gender, DOB, Ethnicity
- 7. Soundex, Gender, DOB, LSOA, Ethnicity
- 8. F_ Soundex, Gender, DOB, LSOA, Ethnicity
- 9. Soundex, Initial, Gender, DOB
- 10 F_ Soundex, Initial, Gender, DOB
- 11. Soundex, Gender, DOB -
- 12. DOB, HSA Centre Residence - FOR POSITIVE STATUS ONLY
- 13. Soundex, HSA Centre Residence - FOR POSITIVE STATUS ONLY
- 14. DOB, PHE Centre Care - FOR POSITIVE STATUS ONLY
- 15. Soundex, HSA Centre Care - FOR POSITIVE STATUS ONLY

The degree of certainty of the match was subjectively classified as “definite” or “partial”. For simplicity, the current paper combines these two types of matches; sensitivity analyses are presented in the main trial report.
